# Cell-Type Specific Features of Circular RNA Expression

**DOI:** 10.1371/journal.pgen.1003777

**Published:** 2013-09-05

**Authors:** Julia Salzman, Raymond E. Chen, Mari N. Olsen, Peter L. Wang, Patrick O. Brown

**Affiliations:** 1 Department of Biochemistry, Stanford University School of Medicine, Stanford, California, United States of America; 2 Howard Hughes Medical Institute, Stanford University School of Medicine, Stanford, California, United States of America; University of Michigan, United States of America

## Abstract

Thousands of loci in the human and mouse genomes give rise to circular RNA transcripts; at many of these loci, the predominant RNA isoform is a circle. Using an improved computational approach for circular RNA identification, we found widespread circular RNA expression in *Drosophila melanogaster* and estimate that in humans, circular RNA may account for 1% as many molecules as poly(A) RNA. Analysis of data from the ENCODE consortium revealed that the repertoire of genes expressing circular RNA, the ratio of circular to linear transcripts for each gene, and even the pattern of splice isoforms of circular RNAs from each gene were cell-type specific. These results suggest that biogenesis of circular RNA is an integral, conserved, and regulated feature of the gene expression program.

## Author Summary

Last year, we reported that circular RNA isoforms, previously thought to be very rare, are actually a pervasive feature of eukaryotic gene expression programs; indeed, the major RNA isoform from hundreds of human genes is a circle. Previous novel RNA species that initially appeared to be special cases, of dubious biological significance, have subsequently proved to have critical, conserved biological roles. An almost universal characteristic of regulatory macromolecules is that they are themselves regulated during development and differentiation. Here, we show that the repertoire of genes expressing circular RNA, the relative levels of circular: linear transcripts from each gene, and even the pattern of splice isoforms of circular RNAs from each gene were cell-type specific, including examples of striking regulation. In humans, we estimate that circular RNA may account for about 1% as many molecules as poly(A) RNA. The ubiquity of circular RNA and its specific regulation could significantly alter our perspective on post-transcriptional regulation and the roles that RNA can play in the cell.

## Introduction

Recently, we were surprised to find that the predominant RNA isoform from hundreds of human genes is a circle, and that circular RNAs were transcribed from thousands of genes in both human and mouse [1]. Circular RNA transcripts had been reported previously for a handful of genes. With the possible exceptions of the circular RNA isoforms of the Sry gene in mouse testis [2] and the *muscleblind* gene in *Drosophila melanogaster* [3] these were generally thought to be rare RNA species, perhaps the result of transcriptional noise. In humans, circular isoforms of the transcripts from the ETS and cytochrome P450 2C24 genes have also been reported; these were found to be inabundant compared to linear RNA isoforms from the same genes [3]–[5]. In recent years, two antisense circular RNAs were discovered and studied more intensely in humans: an antisense transcript from the INK4A-ARF locus, cANRIL, and an abundant antisense transcript to CDR1; the latter was recently reported to be a microRNA sink [6]–[9].

The ubiquitous expression of circular RNA in human and mouse cells has now been independently confirmed by high throughput sequencing of the RNase R treated, ribosomal-depleted fraction of RNA, combined with a previously published informatic algorithm to identify circular RNA [7] as well as by a second report characterizing RNA after ribosomal RNA depletion [8]. In the former report, a large majority of the circular isoforms we had described (1025 of 1319) were also identified by deep sequencing of RNase R-treated RNA. This overlap in circles identified in these two studies is noteworthy because the more recent report focused on fibroblasts, while we previously analyzed RNA isolated from leukocytes and pediatric leukemias.

Here, we describe a more systematic bioinformatic and statistical genome-wide study that significantly expands the catalogue of circular RNAs identified in human cells and reveals significant regulation of circular RNA expression. In many applications, computational challenges associated with mapping and with distinguishing between sequencing errors and sequence homology prevent reliable identification of structural variants, including circular RNAs. Indeed, although de novo splicing detection algorithms have been used in more than a thousand published studies, including studies aimed at identifying gene fusions and internal tandem duplications, most instances of scrambled exons in human RNAs, and thus the circular species that they represent, had eluded detection.

## Results

### Improved detection of circular RNA across diverse cell types

A major challenge in bioinformatic and statistical identification of novel RNA isoforms, particularly circular RNA, involves distinguishing bona fide evidence of scrambled exons in RNA from confounding factors such as sequence degeneracy at exon boundaries and sequencing errors. To address these challenges and to identify circular isoforms from public ENCODE RNA-Seq data, we developed a new bioinformatic approach. The main idea behind our computational method is that it refrains from qualitative hard thresholding of read alignment quality, and instead computes statistical averages of alignment quality scores. This approach allows us to distinguish putative novel splice junctions where the majority of reads align to the ‘novel’ junction with high quality alignment scores from those where reads with high alignment scores are rare. The method allows for systematic FDR-based thresholding, rather than qualitative cut-offs, to determine classification as a scrambled junction at a prescribed confidence level.

Our method was focused on identifying circular RNA transcribed from genes whose linear isoform exons are annotated: we first built a database of all scrambled junctions between annotated exon boundaries, essentially as previously described [1], extending the database to annotated hg19 UCSC ‘knowngene’ exon boundaries. Importantly, we did not impose a lower threshold on the length of annotated exons in our database, instead generating database entries of short annotated exons by an ‘in silico’ rolling circle. We required a minimum of 10 nt on both sides of a diagnostic read to span a scrambled exon-exon junction.

The improved sensitivity of this approach compared to previous methods allowed us to identify thousands of previously unreported circular isoforms and some very small circular RNAs, exemplified by a <150 nt circular RNA isoform ABTB1 resulting from the splicing of two short exons. Other small circular isoforms that we identified and confirmed include a 204 nt circular isoform from a single exon of LINC00340 - a long intergenic noncoding RNA, and a two exon circle of 151 nt from the RNA binding motif gene RBM5.

Experimental and bioinformatic noise can give rise to spurious evidence of circular transcripts, especially for highly expressed genes. To tackle this problem, we combined the bioinformatic approach above with a statistical strategy to distinguish reads supporting exon scrambling from reads likely to be homology and sequencing artifacts. Briefly, we did not impose any thresholds on alignment quality of either read 1 (aligning to a diagnostic scrambled exon-exon junction) or read 2 (aligning to a canonical isoform or a diagnostic scrambled junction). All read pairs with evidence of scrambled exon splicing at a particular pair of genomic coordinates were aggregated by averaging, measuring the overall quality of reads aligning to the putative circle.

**Figure 1:**
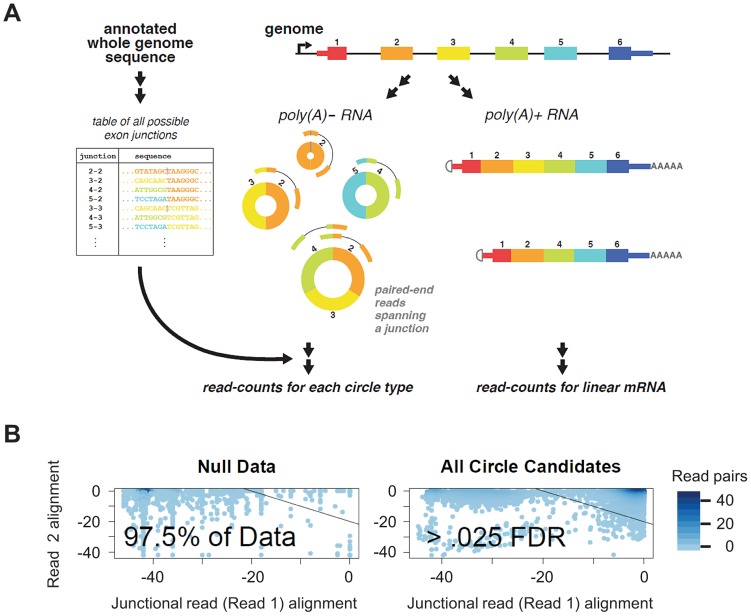
Bioinformatic and statistical method for detecting circular isoforms. A) We created a custom database of all UCSC known-gene annotated scrambled exon-exon junctions. By mapping paired end 76 nt sequencing reads from poly(A) depleted RNA, we detected thousands of distinct circular RNA isoforms, including many cases where multiple circular isoforms are transcribed from the same locus. Our informatic pipeline required that one read (read 1) map to a diagnostic exon x - exon y junction (y< = x) and the other read map within the inferred circular isoform. B) Statistical scores improve filtering: We modeled the distribution of alignment statistics for reads from under an empirical null. Estimating the empirical null distribution of alignment quality for read 1 (required to map to a diagnostic circular exon-exon junction) and read 2 (which need not be junctional) allows us to compute a per-circular isoform FDR and statistically identify artifacts. The bulk of detected circles (illustrated at right) have alignment profiles that distinguish them from those detected under the null model. We used an estimated FDR threshold of .025, shown on plot.

We generated an empirical null distribution of average alignment qualities using “decoy” read pairs. These “decoy” reads had the property that read 1 mapped to a scrambled intragenic exon X – exon Y junction and read 2 mapped within the same gene but would be excluded from a circle composed of exons Y, Y+1, … X, see Figure 1A. These alignment qualities were averaged across all reads for each circular RNA and generated our null distribution of the alignment quality as depicted in Figure 1B. This approach allowed us to compute a per-isoform FDR (by referring the alignment score per isoform to the empirical null distribution) and reduce calls of false positive circular isoforms which riddled the data before this approach was applied.

Using this approach, we were able to enhance statistical discrimination between case 1: false positive evidence of circular RNA isoforms in highly expressed genes resulting from reads with sequencing errors observed due to high sampling of these genes, and case 2: bona fide low-level expression of circular isoforms from these highly expressed genes. For example, the vast majority of reads (99.995%) from putative circular isoforms of GAPDH had a FDR significantly surpassing our threshold of .025; these reads would be taken as evidence of circular isoform expression with a naïve approach.

We applied our method to a large publicly available data set from the ENCODE consortium (see Table 1), with the goal of identifying novel RNA circular isoforms and studying regulation of circular RNA. This dataset consisted of 76-nt paired-end reads from RNA isolated from 15 different cancer and non-cancer cell lines representing mesodermal, ectodermal, endodermal lineages and the pluripotent H1-hESC (see Table 1). Each RNA sample was depleted of poly(A) RNA, size selected to be above 200 nt, and subsequently subjected to ribosomal RNA depletion by RiboMinus (see ENCODE protocols). We have also made use of public analysis of the matched poly(A)-selected samples from these cell lines published by the consortium. Note that the statistics above are absolute counts not corrected for sequencing depth, which varied by sample.

**Table 1:** ENCODE cell types analyzed by lineage and cancer status.

Lineage	Cancer	Non-Cancer
Mesodermal	K562	GM12787
Endodermal	MCF-7	AG04450
	HeLa S-3	BJ
	SK-N-SH_RA	HSMM
		HMEC
		HUVEC
		NHEK
Ectodermal	HepG2	NHLF
	A549	
Pluripotent	H1-hESC	

Across the 15 cell types, at an FDR of .025, we found 46866 distinct intragenic splice junctions at annotated exon boundaries in 8466 genes. Across cell types, we detected the largest number of genes with evidence of circular RNA expression in the leukemia cell line K562 (16559 distinct circle-specific splice junctions); in the fetal lung fibroblast line AG04450, we detected 11590 distinct splicing circle-specific splice junctions and in the human foreskin fibroblast line BJ, we detected 7771 (this is not a typo: [7] reports exactly the same number of circular isoforms). Recently, 7771, 2229 and 485 splice junctions, of ‘low’, ‘medium’ and ‘high’ stringency, respectively, representing circular RNA were identified by another method in Hs68 cells, a human fetal foreskin fibroblast line [7].

### Validation

**Figure 2:**
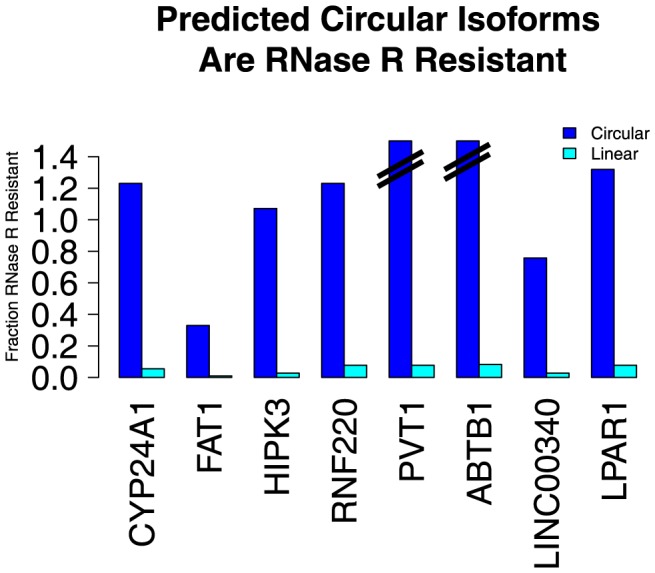
Predicted circular isoforms are resistant to RNase R. HeLa RNA was treated with RNase R or a mock treatment, and then subjected to qPCR with isoform-specific primers. The fraction of linear and circular isoforms was normalized to the value measured in the mock treatment. All tested circular isoforms resisted RNase R, including CYP24A1 (1106 nt), FAT1 (3283 nt), HIPK3 (1099 nt), RNF220 (742 nt), PVT1 (410 nt) and ABTB1 (130 nt). The depletion of the FAT1 circle (the largest circle tested) by RNase R may be due to occasional nicking by contaminating endonuclease activity. We hypothesize that the apparent increase in abundance of some upon RNase R treatment is due to more efficient priming in the RT after linear and ribosomal RNA depletion.

We used the enzyme RNase R, a highly processive 3′ to 5′ exoribonuclease, to test our computational predictions of circular RNA species. This exonuclease is not expected to digest circular RNA because they lack the required free 3′ end but readily digests linear RNAs with a 3′ single stranded region of greater than 7 nucleotides [10]. We tested a panel of 8 putative circular RNAs varying in size, abundance and abundance of the corresponding linear isoforms: ABTB1, FAT1, HIPK3, CYP24A1, LINC00340, LPAR1, and PVT1. As positive controls, we included 3 genes with strong prior evidence of circularization: MAN1A2, RNF220 and CAMSAP1. We treated total RNA from HeLa cells with either RNase R or a mock enzyme treatment. For each sample, we performed an RT with random hexamer primers and used qPCR to quantify the change in abundance of species with scrambled exons compared to species with exons that we predicted found only in linear RNA isoforms, following treatment with RNase R. All the RNA species that we had predicted to be circular were resistant to RNase R whereas all predicted linear sequences were highly sensitive to RNase R (see Figure 2), providing strong evidence that our computational method specifically identifies circular RNA species.

### No evidence of RNA transcription from circular RNA templates

Because the ENCODE libraries were constructed to preserve strand directionality, we could analyze the data for evidence that circular RNAs may serve as an RNA-dependent RNA polymerase as has been shown to occur in some siRNA pathways [11] and in viral or viroid replication [12]. Among paired end reads that support either sense or antisense circular RNA, with a diagnostic circular junction between exon boundaries annotated for linear RNA, we found a strong and significant bias in the directionality of reads (almost 100% of reads from >93% of putative circles). This bias supports the hypothesis that the significant majority of RNA circles formed using splice sites shared with annotated linear RNAs are transcribed from the same strand as the canonical linear RNA. By this analysis, the percentage of circular isoforms in the sense orientation (with respect to the linear isoform) was 96% for HMEC and >99% for the other 14 cell types. This evidence argues against a primary function of circular RNA serving as an RNA template for an RNA-directed polymerase. A small minority of reads had a polarity inconsistent with transcription in the same direction as linear RNA; we believe in most cases these represent artifacts of reverse transcription, perhaps induced by RNA secondary structure. Note that our intention was not to identify un-annotated antisense circular RNA, and we did not search for circular RNAs that might have been produced by splicing a primary transcript complementary to the annotated transcript.

### Relative abundance of circular and linear RNA isoforms

We sought to determine the relative abundance of each circular RNA compared to its cognate linear RNA. This required estimating the relative abundance of each linear RNA, the relative abundance of each circular RNA, and an “equivalence factor” or normalization constant relating the number of RNA molecules represented by 1 measured unit of linear RNA to 1 measured unit of circular RNA. For linear RNA abundance, we used the estimates generated by the public ENCODE consortium analysis of polyadenylated fractions, represented in RPKM units (reads per kilobase of transcript per million mapped reads in the sample). Our estimate of each circular RNA isoform's abundance from sequencing was the number of read pairs, in poly(A)-minus fractions, in which one read spanned a circular junction (note that counting junctional reads in this manner inherently normalizes by gene length). To determine the equivalence factor (the number of junctional read counts expected for a circular RNA expressed at the same level as a transcript with an RPKM of 1), we measured the abundance of circular and linear isoforms of FAT1 and HIPK3 across three ENCODE cell lines (A549, AG04450 and HeLa) by qPCR. This allowed us to relate the abundance of the linear isoforms of FAT1 and HIPK3 as measured in units of RPKM to the abundance of the circular isoforms of these genes as measured in units of junctional read counts. Since the equivalence factor is the same for all genes in the genome, we were then able to compute the relative abundance of circular and linear isoforms for all genes detected in the sequencing data.

FAT1 and HIPK3 were chosen because they have large, abundant circular RNA isoforms and high linear RNA isoform expression, thus mitigating potential factors confounding this estimation such as rolling circle amplification of small circular RNA during the RT, and statistical uncertainty introduced by estimating the expression of low abundance circle or linear isoforms.

These estimates suggested that there was roughly 1 molecule of circular RNA for every 100 molecules of poly(A) RNA in the cell lines we evaluated: A549, AG04450 and HeLa. For roughly 50 genes in each cell line, circular transcript isoforms were estimated to be more abundant than linear isoforms (see Tables S1, S2, S3 for a complete list of the relative linear: circular isoforms per gene genome-wide). For most genes with circular RNA isoforms, the abundance of the circles was roughly 5–10% that of their linear counterparts. At least among this small sample of cell lines, the differences in growth rate and developmental origin do not appear to fundamentally alter the genome-wide rate of circular RNA expression.

**Figure 3:**
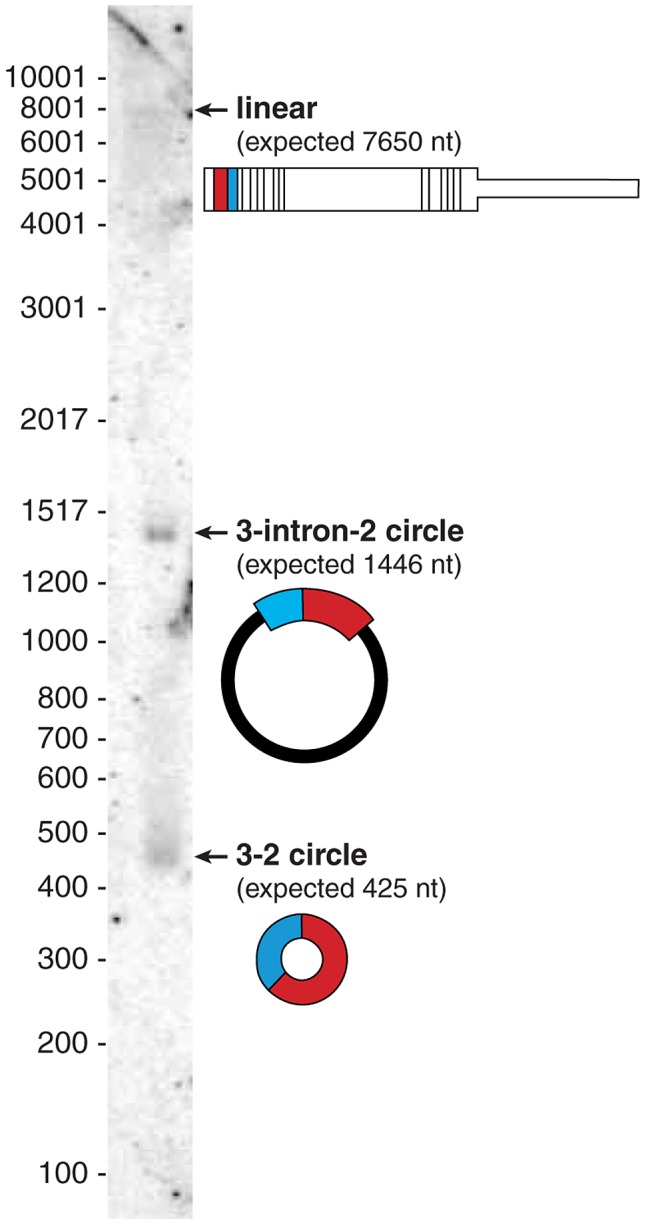
Northern blot shows the dominant isoform of CAMSAP1 is circular. Northern blot on total HeLa RNA probed for exons 2 and 3 reveals three distinct bands: the largest, the canonical linear isoform of CAMSAP1 (∼7800 nt), a 1446 nt representing a circular isoform of CAMSAP1 containing exon 2, exon 3 and the intervening intron; a 425 nt band representing the fully spliced circular isoform of CAMSAP1 consisting of exons 2 and 3.

As a spot-check of our sequencing based estimates of relative abundance of linear and circular isoforms, we performed a Northern blot for CAMSAP1 with total RNA from HeLa cells (see Figure 3). Sequencing based estimates suggested that the circular isoform of CAMSAP1 consisting of exons 2 and 3 was 20 times more abundant than the linear counterpart. The Northern blot shows that CAMSAP1 circular isoforms are more abundant than the linear isoform. Intriguingly, one of the major bands (at 1446 nt) is an unexpected circular isoform consisting of exon 2 - intron 2 - exon 3. RT-PCR bands consistent with both isoforms were amplified from RNase R treated HeLa RNA; Sanger sequencing of the gel-purified bands verified their structure (data not shown).

RNA-Seq also provided evidence for this retained-intron circle: in poly(A) depleted fractions of HeLa-S3, the highest read density was in exons 2 and 3 followed by intron 2, with other introns more than 10-fold lower. Of note, our estimate of the ratio of intron 2 to exon 3 expression (based on number of reads with zero mismatches to the genome) was somewhat higher in the nuclear fraction (38%) compared to in the cytosolic (10%) or RNA isolated from total cells (16%). There was also cell type variation of the ratio of intron 2 to exon 3 read density in “cell” fractions across the ENCODE data set, from 16% in HeLa-S3 down to 3.5% in NHEK, suggesting that intron-retention in CAMSAP1 circles may be under regulatory control.

### RNA circularization does not require long flanking introns

We previously reported that genes with circular RNA transcripts tend to have larger introns than genome-wide averages. That analysis showed that even after controlling for the tendency for intron lengths to decrease from 5′ to 3′ along the canonical transcript [1], [13], the introns immediately flanking the exon boundaries that participated in the scrambled splice were significantly longer than average. Here we further investigated the relationship between intron length and circularized exons in this deeper survey of circular RNA expression. For each UCSC annotated gene, for each annotated splice site, we defined the flanking intron length for the 3′ and 5′ splice sites as the distance to the nearest upstream or downstream 5′ or 3′ splice site respectively. To control for systematic biases, for example, that genes expressing circular RNA isoforms have relatively large introns compared to genome-wide averages (as we have found previously), we performed the following analysis.

**Figure 4:**
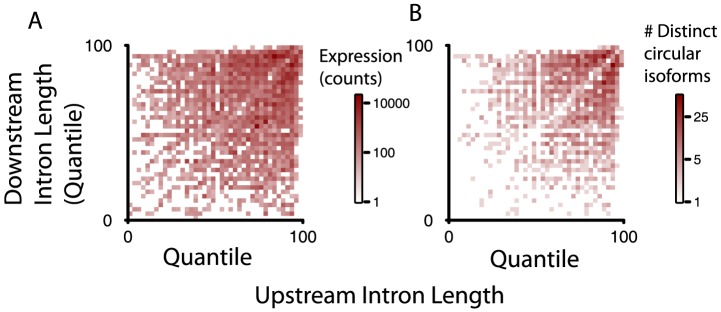
Intron length is enriched around exons defining circular RNA, but alone not explanatory of circular RNA expression. Intron lengths flanking circular isoforms are calculated as described in the main text. A) and B) show the genome-wide distributions of flanking intron length, normalized by their quantile rank within a gene (shortest = 0; longest = 100); A) weights each isoform by total reads summed over all replicates and samples; B) counts each isoform once, provided it has at least 20 distinct read counts.

We ranked introns that flanked spliced exons generating circular RNA isoforms in two ways: 1) weighting the lengths of introns flanking each circular isoform by the abundance of the corresponding circular RNA (right panel in Figure 4); 2) counting each circular isoform once regardless of its expression level (left panel in Figure 4). For the analysis depicted in Figure 4, for each gene, we ranked the length of each intron according to its length. We then converted each rank value to a quantile: for example, the second largest intron in a gene with 5 introns would receive a quantile of 40 ( = 2/5 * 100%). For reference, under a null model where the rank of intron length had no relationship with propensity to flank a circular splice donor or acceptor, the heatmaps would have uniform intensity regardless of the quantile represented. We found that the relative length of the flanking intron did not reliably determine which exons were spliced to form an RNA circle, although within a gene, longer introns were more likely to flank circularized exons.

To test whether small variations in intron length might explain the dynamic range in intron length quantiles observed in Figure 4, we also examined the relative length of each intron flanking a diagnostic donor or acceptor site in the circle as a fraction of the largest intron length in the gene (Figure S1). Thus, if one of the introns flanking a splice site diagnostic of a circle were the longest intron in the gene, its ratio compared to the maximum intron length would be 1. The null distribution we considered was based on the relative length of the second vs. third largest intron in the set of genes evaluated. Unexpectedly, we found that, measured as a fraction of maximum intron length, introns flanking circular junctions were, on average, smaller than expected from the null distribution, perhaps explained by a single atypically long intron within genes expressing circular isoforms.

### Regulation of circular RNA isoforms

**Figure 5:**
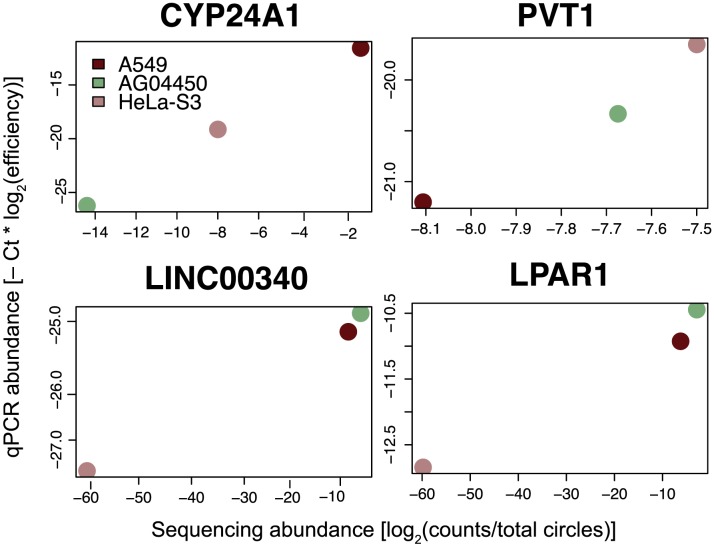
qPCR validation of relative circular RNA expression across cell type. Total RNA from A549, AG04450 and HeLa cells was probed by qPCR using primers specific for circular isoforms of the indicated genes, and abundances were normalized using primer efficiencies estimated with a dilution series. Sequencing-based estimates are shown by comparison; sequencing values are depicted as a log fraction of total circle counts per experiment. Each qPCR and sequence value is calculated from the average of two biological replicates. Expression of LINC00340 and LPAR1 in HeLa is not detectable with the sequencing depth in this data, and these values were pinned at −60 on the log scale.

We explored regulation of circular RNA expression using the ENCODE RNA-Seq data for a number of cultured cell lines, then did an independent evaluation of some of the identified circle expression variation using qPCR (see Figure 5). Some of the genes we tested by qPCR (CYP24A1, PVT1 and LPAR1 and LINC00340) expressed circular RNA isoforms that were predicted from sequence data to vary across cell lines; others (FAT1, HIPK3) appeared from the RNA-Seq data to have constant levels of circular isoform expression in A549, AG04450 and HeLa cells (data not shown).

To assess variation in circular RNA expression genome-wide, we estimated the abundance of circular RNA from sequence data based on the diagnostic splice-junction counts described earlier; estimates of linear transcript abundance were from the ENCODE consortium's analysis of poly(A) gene expression. For a set of relatively highly expressed circular isoforms, we evaluated the fit of a Poisson model in which circular RNA expression increased with linear isoform abundance, controlling for effects of sequencing depth and incorporating experimental variation by treating experimental replicates as distinct. We also included cell-type effects to further account for circular RNA expression.

We observed the largest dynamic range in circular RNA production in the gene CYP24A1, a candidate oncogene encoding a component of the vitamin D3 metabolic pathway. Its linear mRNA product and the CYP24A1 protein have been reported to be expressed at elevated levels in many primary lung and other cancers, and in many lung cancer cell lines, including A549; no amplification of the CYP24A1 gene has been reported in A549 [14]–[19], although CYP24A1 is frequently amplified and mutated in primary human cancers [20].

**Figure 6:**
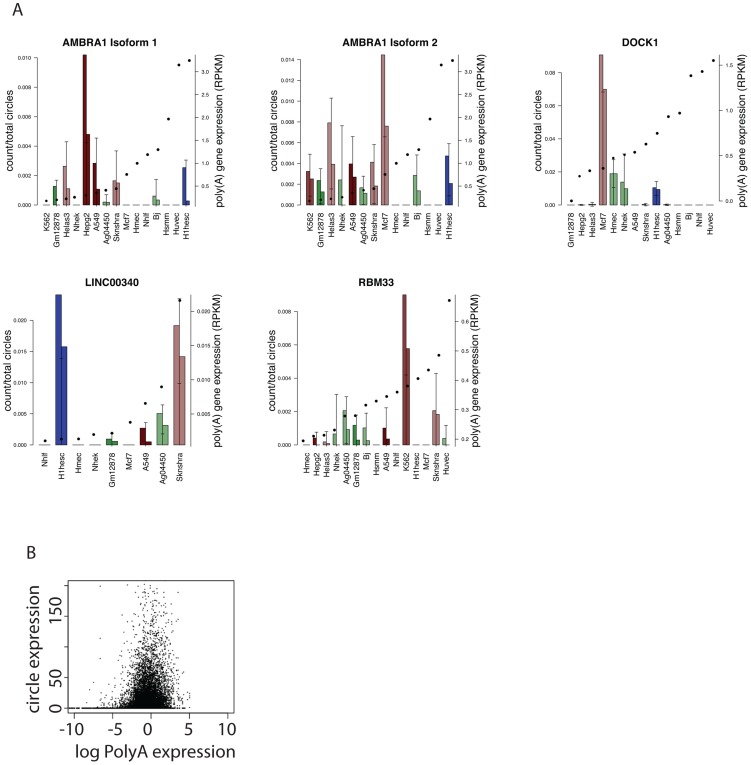
Quantitative regulation of circular to linear isoform ratios. A) Examples of circular RNAs with cell-type dependent expression as predicted by a genome-wide statistical model. Circular isoform abundance was estimated as a fraction of total circular RNA expression per replicate, and error bars represent statistical variation (3.5 sd of the mean); replicates are depicted separately. Two circular isoforms of AMBRA1 are shown. For each gene, cell types are ordered left to right by monotonic increasing expression of the linear isoform as measured by RPKM, with RPKM value overlaid as a solid dot. Bar plot colors are consistent across cell types: red representing cancer cell lines, blue H1-HESC and greens are non-cancers; shading from dark to light representing endoderm, mesoderm and ectoderm. B) Across cell lines, no genome-wide trend between circle expression and linear transcript expression as measured by log RPKM.

Our statistical model also suggested that other highly expressed RNA circles had cell-type specific increases in expression that could not be accounted for by an increase in overall expression of the corresponding linear RNA. One example is the circular isoform of DOCK1, whose linear isoform encodes a “dedicator of cytokinesis”, a RacGEF, and was the most highly expressed circular isoform in MCF-7, a breast cancer cell line. DOCK1 also had the highest estimated ratio of circular: linear RNA expression in MCF-7 among all cell types in the ENCODE panel, including those where the linear isoform of DOCK1 was more highly expressed (see Figure 6).

Figure 6 depicts other examples of genes with cell-type-specific selective increases in the ratio of circular to linear RNA isoforms. One example was the much higher expression of a circular RNA isoform of RBM33 in K562 cells compared to the other cell types. We have previously detected RBM33 circles in human leukocyte and leukemia samples and mouse brain [1], suggesting the possibility of evolutionary conservation. RBM33 has not been extensively studied, but duplication of a locus including Sonic Hedgehog and RBM33 has been associated with congenital muscular hypertrophy [21]. Similarly, expression of a circular isoform of the long intergenic noncoding RNA LINC00340 was specifically elevated in H1-hESCs. In H1-hESCs, sequencing data suggested that the circular isoform of LINC00340 was the fourth most highly expressed circular RNA of all detected circular isoforms. Circular isoforms of other LINC RNAs, including LINC00263 and LINC00265, were also identified in our analysis. Because noncoding RNAs, including LINC RNAs are generally less well annotated than messenger RNAs, it is possible that our analysis may still have under-detected circular isoforms of these RNAs as we did not specifically attempt to improve their representation in the UCSC knowngene annotation.

A final highlighted example of cell-type-specific selective increases in the ratio of circular to linear RNA isoforms in Figure 6 is AMBRA1. Two different circular isoforms of AMBRA1 RNA were differentially regulated in MCF-7 and HepG2 cells. MCF-7 cells expressed higher levels of a longer isoform (362 nt) while a shorter isoform (182 nt) was more highly expressed in HepG2. AMBRA1 plays a key role in autophagy; deficient mice have excessive cell death by apoptosis [22]–[23].

In these specific examples, and in general, variation in the abundance of hundreds of circular RNA isoforms appeared to have little or no correlation with variation in the abundance of the cognate linear RNA between the cell lines we compared. In particular, we did not observe a correlation between circle-specific junctional counts and overall abundance of the corresponding RNA as measured by RPKM, even at the lowest levels of gene expression. Further evidence that RNA circles are not just an accidental aberration of normal splicing is provided by the fact that circular RNA isoforms are generated by splicing very specific pairs of exons (see discussion below).

### Circular RNA isoform splice site selection varies across genes

**Figure 7:**
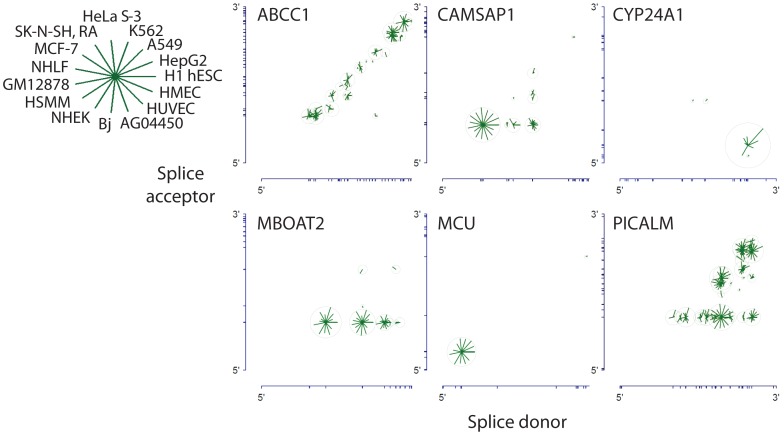
Circular isoform expression patterns involves a variety of splicing patterns including proximal pairing and combinatorial expression. Gene structures are represented along the axes with tick marks at splice site boundaries. Each circle is centered at the genomic coordinates corresponding to the donor and acceptor splice sites of the detected circular isoform. The length of the line segment is proportional to the log of the expression level of the circular isoform; the ring represents the maximum expression of the circular isoform across cell types. CYP24A1 and MCU exhibit striking expression preference for a single circular isoform. MBOAT2 exhibits a strong preference for a single splice acceptor site paired with multiple donors. ABCC1 exhibits preference for proximal pairs of splice donors and acceptors; CAMSAP1 exhibits a strong preference for either a particular single acceptor and or a particular single donor, whereas PICALM is an example of a gene with high combinatorial use of splice sites.

When a gene encodes multiple alternatively spliced circular isoforms, what patterns characterize the use of splice acceptor and donor pairs to produce the circle junction? To study this question, we distinguished three broad classes of splice site pairings, which we term stereotyped, proximal and combinatorial pairing, respectively. Examples of each are depicted in Figure 7. For most genes that have circular RNA isoforms (the “stereotyped” class), a single splice site donor and acceptor pair were either used exclusively or strongly preferred in the splice that gave rise to the circular isoforms of the gene; this was the case, for example, for CYP24A1 and MCU. While CYP24A1 was the most highly expressed circular RNA among the examined cell lines and MCU was among the 20 most highly expressed circular RNAs in 9 different cell types, only one circular splice variant from each gene was overwhelmingly preferred (see Figure 7).

A variant of stereotyped splicing was exemplified by the circular RNA isoforms of MBOAT2. These isoforms were expressed at levels similar to MCU, but with a distinctly different pattern of splicing: one particular splice acceptor was highly preferred, but several alternative splice donors were used and each produced similar levels of the corresponding spliced circular RNA isoform. It is noteworthy that none of the exons that participate producing the MBOAT2 circles have been reported to participate in alternative splicing of linear RNA MBOAT2 isoforms.

For many transcripts in which multiple splice donor and multiple splice acceptor sites were used in circular splicing, proximal donor-acceptor pairs were strongly preferred. This “proximal” pattern of circular splicing is exemplified by the circular isoforms of ABCC1.

The “combinatorial” pattern of circular splicing is exemplified by CAMSAP1 and especially PICALM. Multiple splice donors and multiple splice acceptors participate in production of circular isoforms, with little preference for proximal donor and acceptor sites. In contrast to PICALM, across cell types, CAMSAP1 has a single dominant isoform.

Although detection of rare circular RNA isoforms increased with sampling depth of the RNA sequences (see Figure S3), within a gene, our data did not fit a simple model where overall expression of circular RNA isoforms predicted the diversity of circular RNA isoforms expressed (Figure S4). Figure S4 depicts other features of intragenic circular RNA splicing patterns across all genes: the majority of genes with detectable circular RNA expression had detectable levels of more than one circular isoform. Also in such genes, the number of splice donor and acceptor sites used in circular splicing was correlated: when more acceptor sites were used in circular RNA products from a particular gene, so were more donor sites.

Considering all genes with circular RNA isoforms, we found that cells generally expressed a small fraction of the number of circular RNA isoforms that could, in principle, be produced by splicing a downstream splice donor to an upstream splice acceptor (see Figure S4C). We quantified this fraction by defining a combinatorial index *C*, which compares the number of observed circular isoforms to the number of possible pairings of the *detected* acceptor and donor splice sites (see Methods). In general, regardless of the total expression level of circular RNA isoforms, half or less of the combinatorial space of circular isoforms—conditioned on acceptor donor and acceptor sites used in circular RNA splicing– had detectable expression, and many genes expressed the minimum number of potential circular RNA isoforms represented by the lowest value of *C*.

### Circular RNA isoform splice site selection is regulated

We used a statistical model to identify genes with regulated use of donor and acceptor sites characterizing the diagnostic non-canonical exon junction. For each gene and each cell type, we estimated the frequency with which each donor and acceptor splice site was used (see Tables S4, S5), and computed confidence intervals for the use of each site by cell type.

**Figure 8:**
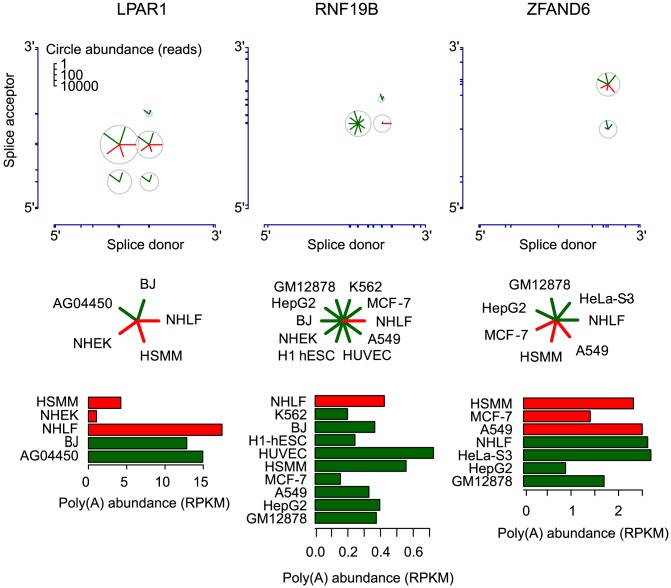
Circular isoform expression is regulated within individual genes. Circular isoform expression across cell types is shown for LPAR1, RNF19B, and ZFAND6 as in Fig. 6. The barplots depict expression of the corresponding linear isoforms in RPKM units. Cell types are colored as red or green to highlight distinct patterns of circular isoform expression. Differential circular RNA isoform expression in LPAR1, RNF19B and ZFAND6 cannot be explained by differences in expression level of polyadenylated transcripts of these genes or sampling depth.

For hundreds of genes, we found statistical evidence of cell type-specific preferences in patterns of splice donor and acceptor usage in the biogenesis of circular RNA (Tables S4, S5). Three of these genes are shown in Figure 8. The RNF19B gene provides a simple and striking example. The only circular isoform of RNF19B RNA detected in NHLF was undetectable in any of the other cell lines examined. Conversely, the dominant circular RNF19B isoform in the other cells was undetectable in NHLF (see Figure 8). In a second example, a single splice acceptor was used in all circular LPAR1 RNAs identified in NHEK, NHLF and HSMM cells, whereas three different splice acceptors were represented in the circular LPAR1 RNAs found in two fetal fibroblast cell lines, AG04450 and BJ. The differences in diversity of circular isoforms were not explained by cell-type specific differences in overall LPAR1 expression.

ZFAND6 is a third example of a gene with regulated circular RNA expression. A549 cells expressed a single circular isoform, while the remaining cell types expressed two circular isoforms. These differences cannot be readily explained either by differences in sequencing depth, cell-type-specific differences in linear or circular RNA expression or any cell-type independent differences in the RNA, such as intron lengths or structure (see Figure 8). For example, among all the cells we examined, NHLF expressed the second highest levels of linear ZFAND6 RNA, but circular ZFAND6 RNAs were undetectable in these cells. Further, we do not observe any correlation between canonical alternative splicing and circular RNA splice site use or patterns in the three genes depicted in Figure 8 (see Figure S2).

### Evolutionary conservation of RNA circles across species, including non-coding RNA loci

To further assess evolutionary conservation of circular RNA expression across model organisms, we surveyed circular RNA expression using published RNA-Seq data from *Drosophila* brains [24]. This analysis revealed hundreds of genes encoding circular RNA isoforms in fly, including abundant expression of a previously described circular RNA isoform from the *muscleblind* locus [3]. *Muscleblind* was among the most highly expressed circular isoforms, but our analysis indicated that circular RNAs from 11 other genes in these samples were even more abundant: the most highly expressed putative circular RNAs were from *Pka-C3*, encoding a cAMP-dependent protein kinase, and *scarecrow* (*scro*), encoding an NK-2 homeobox protein. Other highly expressed RNA circles included *Caps, ps, mGluRA, caps, snap25, jp, zfh2* and two genes of unknown function, CG40178 and CG17471. Overall, we found evidence for exon scrambling in more than 800 distinct *Drosophila* splice junctions supported by more than one sequencing read (Table S6).

Additional evidence supporting some evolutionary conservation of circular RNAs is found by considering mouse genes represented in brain RNA-Seq data [1]. Genes whose human orthologs expressed circular RNAs were statisteically more likely to have evidence for circular isoforms in the mouse RNA-Seq data. Roughly 4% of genes with expression in both mouse and human data and which encoded orthologous proteins also encoded circular RNA detected in both data sets compared to a null expected rate of 2.5%. This suggests modest conservation of circular RNA expression from loci with orthologous protein products, ignoring finer features that might influence conserved expression of circular RNA, such as developmental stage. In addition, several genes encoding exclusively non-coding RNA species, including IPW (Imprinted in Prader-Willi syndrome) and the oncogene PVT1 were expressed as circular isoforms in both mice and humans.

## Discussion

Characteristic changes during development and differentiation are a pervasive feature of global gene expression programs. We systematically searched for evidence of circular RNAs in a large corpus of RNA-Seq data generated by the ENCODE consortium as well as in RNA-Seq data from *Drosophila* brain. We found that circular RNA comprises a significant fraction of cellular RNA and that the relative abundance of circular isoforms and the specific patterns of splice site usage in RNA circularization are regulated in a gene-specific and cell-type specific manner. The results strongly suggest that circular RNAs are a common, abundant and potentially developmentally regulated component of the gene expression programs in diverse animal species.

To improve our sensitivity and specificity in detecting circular isoforms, we developed improved bioinformatic and statistical methods that enabled more reliable discrimination between bona fide evidence of exon scrambling and artifacts introduced by high throughput sequencing and sequence homology within a gene. This improved performance allowed us to detect a more extensive catalog of circular RNA than previously reported, including small RNA circles, RNA circles formed by non-canonical splicing of short exons and noncoding RNAs. Improved detection of circular RNA isoforms has also allowed us to characterize the extent of differential circular RNA splicing within a single gene, and to study variation in alternative splicing of circular RNA; indeed, this method may have wider applicability in the discovery of novel RNA splice junctions and detection of other variant sequences.

qPCR quantification and extensive analysis of RNA-Seq data has allowed us to estimate that the number of circular RNA molecules is roughly 1% of the number of poly(A) molecules in the cells we investigated. This estimate is remarkably similar to a report published more than 30 years ago, which found physical evidence of circular RNA by examining cellular RNA by electron microscopy [25].

We tested the hypothesis that circular RNAs might be the result of a background “noise” level of dysfunctional splicing. Under this model, we would expect a positive relationship between linear RNA isoform expression from a given gene and the probability of detecting a circular RNA isoform from that gene. We found no evidence of such a relationship, suggesting instead that even low rates of circular RNA production are regulated, or that highly expressed genes have evolved specific mechanisms to prevent splicing errors that could result in RNA circles.

Our initial report of the ubiquity of circular RNA, based on sequencing an ribosomal-RNA depleted RNA fraction, has since been confirmed in an independent study in which circular RNAs from human and mouse fibroblasts were enriched by treating RNA with RNase R, and in a second genome-wide search for evidence of circular RNA by sequencing ribosomal-RNA-depleted RNA samples [7], [8]. The analysis presented here significantly expands the catalog of circular RNAs expressed by humans and *Drosophila*. It is likely that human cells express even more circular RNAs than we report here: we did not attempt a ‘de novo’ identification of circular RNA, and instead focused on circular RNA produced by splicing at annotated exon boundaries.

For example, by analysis of a Northern blot for CAMSAP1 in HeLa cells, and a subsequent limited bioinformatic survey of 6 genes, we found evidence of cell-type specific variation in rates of intron retention as well as evidence that circular, intron-retained transcripts in HeLa-S3 cells may be sequestered in the nucleus and potentially exported to the cytoplasm. CAMSAP1 is a calmodulin regulated gene and has conserved circle expression in mouse and *Drosophila* (spp4), and it would be interesting to study intron retention in these organisms.

The most highly expressed circular RNA identified in our analysis was from the CYP24A1 gene, in a lung cancer cell line, A549. CYP24A1, which encodes 1,25-dihydroxyvitamin D 3 24-hydroxylase, has been suggested to play a role in the pathogenesis of many carcinomas [14], [18], [26]–[32]. We found that the circular isoform was expressed at levels comparable to the canonical linear form. Although the circular isoform of CYP24A1 (which includes all but the first and last exons of CYP24A1) could in principle encode a protein lacking the N terminal mitochondrial localization signal, we have not found evidence for such a protein by mass spectrometry on A549 cell lysates (unpublished data). This finding is consistent with other evidence that despite the formal possibility of translation of circular RNA by the eukaryotic ribosome [33]–[34], circular RNAs do not in general act by encoding a protein.

Recent reports have shown that an antisense circular transcript from the CDR1 locus is enriched for functional microRNA binding sites [7], [8]. However, in a preliminary analysis, we have not found evidence that enrichment of microRNA binding sites is a global feature under selection in the sequence of the thousands of circular RNAs profiled in this paper. For example, we see a roughly 5% enrichment of microRNA binding sites in a 66 nt window in sequence flanking diagnostic circular RNA junctions in circular RNAs which are highly expressed in at least one cell type compared to the number of binding sites in the junctional sequences flanking all detected circular RNA junctions (see Figure S5). The low enrichment is perhaps not surprising considering that the vast majority of the transcripts we surveyed in [1] and in this report are transcribed in the same sense with respect to the linear mRNA isoform. Therefore, except for ‘diagnostic’ junctional sequence and secondary and higher order structure, circular and linear isoforms would have the same potential to bind microRNA, albeit with a different degree of stability. Certainly, the potential genome-wide interplay between microRNAs and circular RNAs warrants further experimental and computational investigation.

Our findings that mouse orthologs of human genes with circular RNA products are themselves more likely to encode circular RNAs are consistent with a similar independent analysis of circular RNA conservation and support the hypothesis that circular RNAs have an evolutionarily conserved function [7]. Thus, although the abundance, ubiquity, and potential developmental regulation of circular RNAs all point to the possibility of important functional roles, their nature and mechanisms are still to be discovered.

## Materials and Methods

### Data used

Raw fastq files available on Sept. 3, 2012 were downloaded from the ENCODE project website and processed in batch using custom Perl scripts. At that time, 2 replicates from each of 15 cell types were available, with the exceptions that 1 HMEC and 3 NHEK were downloadable. We selected all long poly(A) minus reads banked at http://hgdownload.cse.UCSC.edu/goldenPath/hg19/encodeDCC/wgEncodeCshlLongRnaSeq. Read 1 and Read 2 reflect directionality of original RNA and were not processed symmetrically.

### Identification of circular RNA

We constructed a custom database for sequence alignment as follows: all UCSC annotated exons in scrambled order were identified and for each pair, 66 nt from each side the 3′ and 5′ ends of flanking exon were concatenated. Sequence alignment of a 76 nt read hence required alignment with a minimum of 10 nt overhang. Cases where exons were <66 nt were treated separately, by concatenating exons upstream of the donor or downstream of the acceptor exon in the scrambled pair, or using ‘in silico’ rolling circle concatenation in cases where the total circle size was smaller than 132 nt.

In detail, read 1 and read 2 were not treated symmetrically as the input library was a directional RNA-Seq library. Read 1 was aligned to UCSC knowngenes and the human genome under bowtie2 default conditions [35]. Reads failing this alignment were aligned to a custom database of all scrambled exon-exon junctions for each UCSC knowngene isoform, again under bowtie2 default conditions. We culled the mate of each aligned read 1, and performed an alignment of this subset of reads to the above UCSC knowngenes and to the custom database of scrambled exon-exon junctions: (thus, in principle, we could have analyzed the data as described above focusing on read 2 and increased the number of detected junctional reads).

In conjunction with the alignment to the above database of exon-exon junctions, we modeled the null distribution for rates of mismatch of reads aligning to this database using a method that should be of general interest in discovery of structural variants using high throughput sequencing data. In overview, we considered all reads that aligned with qualities a) and b) below, without imposing hard thresholding on the quality of alignment of either read:

- Read 1 maps to an annotated hg19-UCSC-knowngene intragenic scrambled junction database. This database contains sequences of length 132 and hence requires a 76 nt read to align with at least a 10 nt overhang.
- Read 2 maps to the same annotated UCSC gene, either to anywhere within the gene or to the same exon-exon junction as read 1.

A read reflecting a circular RNA isoform transcribed from the same strand as the canonical isoform has the property that read 1 maps to the −orientation and read 2 to the +orientation.

Alignment scores were calculated using the bowtie2 default which for example, adds a ‘−6’ penalty for a mismatch between the reference and aligned read at a high quality base call. Summing penalties for mismatches produces an overall alignment score per read, one score for the read spanning junction (read 1) and one score for read 2. Three statistics measuring alignment were calculated for each pair of scrambled exons for each UCSC isoform supported by at least one read: the average alignment score of read 1, the average alignment score of read 2, and the average product alignment score of read 1, read 2 although this measure was not ultimately used to calculate the FDR reported.

In detail, to compute the FDR, we created a null distribution of the joint alignment statistics for read 1 and read 2 using reads where read 1 mapped to a junction between exon x and exon y (x> = y) and read 2 mapped upstream of exon y or downstream of exon x which is incompatible with it deriving from a circular RNA molecule. We used the pair of (read 1, read 2) alignment statistics from such reads to generate the FDR per isoform depicted in Figure 1.

Subsequently, all reads were filtered to an FDR level of .025 unless otherwise specified. See Table S7 for a complete list of scores.

### Tests of RNase R resistance

HeLa total RNA was isolated by TRIZOL lysis followed by PureLink purification of the aqueous phase (Life Technologies). 2 micrograms of total RNA was treated in a 10 microliter reaction with 0 units (mock treatment) or 20 units of RNase R (Epicentre) in 1× RNase R buffer, 1 unit/microliter murine Ribonuclease Inhibitor (New England Biolabs), and incubated at 37DEGC for 1 hr. 1 microliter 1 mM EDTA, 1 microliter 10 mM each dNTP, and 1 microliter 100 microM random hexamer were added and the RNA denatured at 65DEGC for 5 min and placed on ice. 4 microliters 5× buffer (250 mM Tris-HCl pH 8, 125 mM KCl, 15 mM MgCl_2), 1 microliter murine Ribonuclease Inhibitor (40 units/microliter), and 1 microliter Superscript III (LIfe Technologies) were added; this cDNA reaction was incubated at 25 deg C 10 min, 50 deg C 50 min, 55 deg C 10 min, 85 deg C 5 min, 4 deg C hold. 0.5 microliter cDNA reaction was used as the template for qPCR and fraction resistant was computed as 2∧(RNase R C_t - Mock C_t).”

### RT-qPCR analysis

HeLa-S3, A549 and AG04450 cells were grown in standard media and conditions. RNA was harvested by lysing cells with the TRIZOL reagent and purified on Purelink columns under ethanol concentrations that retain small and large RNAs. Total RNA reaction was reverse transcribed using the SuperScript III First-Strand Synthesis System (Life Technologies, Carlsbad, CA) with random hexamers according to the manufacturer's instructions. 500 ng/ul of cDNA was then used for each qPCR validation; dilution series were performed at concentrations of .5, 5, 50 and 500 ng/ul. Each qPCR experiment was done in biological duplicate with 3 technical replicates each.

### Computation of moles of circular compared to Poly(A) RNA

For each cell type, we downloaded gtf files with gene level RPKM (Reads per kilobase mapped) estimates from the Poly(A) fraction; eg. for SK-NS-H_RA, we downloaded the file: http://hgdownload.cse.UCSC.edu/goldenPath/hg19/encodeDCC/wgEncodeCshlLongRnaSeq/wgEncodeCshlLongRnaSeqSknshraCellPapGeneGencV7.gtf.gz

RPKMs were summed across all genes to estimate total annotated poly(A) transcript abundance. In parallel, we summed all reads mapping to a circular RNA junction. These two values provide total abundance estimates of poly(A) and circular RNA respectively, up to a normalizing constant. We determined that normalizing constant by performing qPCR with two calibrating genes: FAT1 and HIPK3. These genes were chosen for the reasons described in the main text. Standard curves were computed for each set of primers listed in Table S8 and used to compute relative expression of linear and circular RNA at the log scale. The difference between the log base 2 of calculated junctional circle counts and log base 2 RPKM and these differences was averaged for the 2 genes, and raised to the power 2 in order to normalize measurements. To test robustness of our estimates, we also performed the above analysis by imposing a filter on circles that could contribute to our estimate of total circle mass. That filter required a circular isoforms have greater than 5 counts in the cell type under consideration. This provided a conservative estimate of the moles of circular vs. poly(A) RNA. Using this filter, we obtained estimates of .6%, 2% and .6% for HeLa, A549 and AG04450 respectively, and is consistent with what we estimate without this filter.

### Modeling of linear to circular expression

For each circular isoform represented by at least 50 counts in one sample (and satisfying an FDR cut-off of .025), we fit a Poisson model per gene modeling circle counts by poly(A) gene expression (genexp), celltype and total circles (totcircles) using the glm poisson model in R with the formula:

cir∼log(genexp)+celltype+totcircles−1. Coefficients in this model were used to choose genes shown in Figure 6 and the table of values is listed in Table S9.

### Northern blotting

10 micrograms total RNA was denatured with glyoxal and run on a 2% agarose gel [36], followed by alkaline capillary transfer onto Brightstar-Plus nylon membrane (Ambion). 32P-labeled probe was generated by random-priming (Prime-It II kit, Stratagene) of a PCR product corresponding to exons 2 and 3 of CAMSAP1 (nt 161–423 of GenBank # NM_015447.3) and hybridized in modified Church buffer (0.5M sodium phosphate pH 7.2, 7% SDS, 10 mM EDTA) at 65DEGC for 16 hr. After washing, the blot was visualized by phosphorimaging (Typhoon, Molecular Devices).

### Isoform specific variation

The following procedure was used to access statistical significance of the use of donor and acceptor sites across cell types. We analyzed donor and acceptor sites separately. Splice sites represented by more than 50 counts (and satisfying an FDR cut-off of .025) in at least one cell type were analyzed using this approach. For each such donor and acceptor site that was supported by more than 5 reads and present in at least two cell types, we computed an exact .999 binomial confidence interval for its probability of use in that cell type. Sites with at least one pair of non-overlapping confidence intervals were identified and used to choose genes depicted in Figure 8. Cell types were collapsed over replicates. A table of all confidence intervals by site and cell type is listed in Table S4.

### Detection of scrambled exon-exon junctions in *Drosophila* and orthology computation

Poly(A) depleted RNA isolated from Drosophila brain, available at ftp://ftp-trace.ncbi.nlm.nih.gov/sra/sra-instant/reads/ByStudy/litesra/SRP/SRP007/SRP007416/ was aligned to a custom database of annotated Drosophila exon-exon junctions using Jan. 2012 flyBase exon annotation and previously described methods and filters. A complete list of detected events is listed in Table S6.

To access evolutionary conservation, orthology of protein products was defined by Inparanoid using the following databases: http://inparanoid.sbc.su.se/download/7.0_current/sqltables/

For statistical assessment of expression of circular isoforms between mouse and human, a list of orthologous genes expressed (measured by detected gene expression from RNA-Seq data sets used to measure circle abundance) was compiled (a total of 1402 genes). We then counted the number of genes in this table with more than 1 circle count in the mouse RNA-Seq data and with expression in the top 100 most expressed circular isoforms in one ENCODE (human) experimental replicate. 57 genes matched this criterion (4%). 147 genes on the list of 1402 were in the top 100 most expressed circular isoforms in one experimental replicate; 332 had more than 1 count in the mouse RNA-Seq data. Under an independence model, we expected 35 genes to match the joint criterion (2.5%). The residual from a chi-squared test for the independence model is (O-E)/sqrt(E) = 3.7, which corresponds to a one sided p value of .0001.

### Analyses of intron lengths flanking circularization splice sites

Read counts were summed across cell types and replicates for each isoform, defined here as a unique combination of gene, circularization splice donor coordinate, and circularization splice acceptor coordinate. Isoforms were filtered by requiring 20 or more read counts in total across ENCODE cell lines. Intron length was computed as described in the text. For each gene, intron lengths were considered either as fractions of the longest intron length within the gene, or as quantile ranks within the gene.

### Analyses of circular isoform expression by cell type

Read counts were summed across replicates for each unique combination of cell type, gene, circularization splice donor coordinate, and circularization splice acceptor coordinate.

### Analyses of circular RNA properties by gene

Read counts were summed across cell types and replicates for each isoform, defined here as a unique combination of gene, circularization splice donor coordinate, and circularization splice acceptor coordinate. Genes where some annotated isoforms satisfied splice acceptor<donor and other isoforms satisfied donor<acceptor were removed from consideration; the remaining genes were then oriented such that all isoforms were acceptor upstream of donor.

For each gene, the combinatorial index *C* compares the number of observed circular isoforms to the number of possible pairings of the detected acceptor and donor sites; *C* = 1 means that all possible pairings were actually detected, whereas *C* = 0 means that the minimum possible number of pairings was detected (we adopted the convention that *C* is undefined when max. poss. isoforms = min. poss. isoforms). Precisely, *C* was defined for each gene as (# of distinct circular isoforms detected – min. poss. isoforms)/(max. poss. isoforms – min. poss. isoforms), where min. poss. isoforms = max(# of distinct acceptor sites detected, # of distinct donor sites detected), and max. poss. isoforms = # of combinations of 1 detected acceptor and 1 detected donor in which the acceptor is upstream of the donor. *C* is evaluated in Figure S4.

### Analyses of total circular RNA expression and number of circular isoforms in individual replicate samples

For each cell type and replicate, isoforms were defined here as unique combinations of gene, circularization splice donor coordinate, circularization splice acceptor coordinate, read 1 orientation, and read 2 orientation.

### Analyses of circular isoform strand orientation

Instances in which the junction-defining read (“read 1”) and its mate-pair read (“read 2”) were on the same strand were removed from consideration. For each cell type, replicates were pooled, and isoforms were defined here as unique combinations of gene, circularization splice donor, circularization splice acceptor, and read 1 orientation. The percentage of circular isoforms in the sense orientation (with respect to the linear isoform) is 96% for HMEC and >99% for the other 14 cell types.

### Analysis of microRNA binding sites

We downloaded a list of all high confidence microRNAs (mature.fa from http://mirbase.org/ftp.shtml annotated as ‘Homo sapiens’) and corresponding 6mer seed match (nt 2–7). For each possible non-canonically ordered exon X, exon Y pair within a transcript in the UCSC knowngene transcript database (enumeration beginning at 0) to 30, we generated a corresponding 132 nt sequence consisting of 66 nt upstream and 66 nt downstream of the exon-exon junction. For each group of exon X -exon Y sequences, we compared the number of microRNA seed matches per offset (from 0 to 126) divided by the total number of junctions evaluated. We compared these statistics for circular junctions expressed at rank <1000 in at least one cell type, ranked based on aligning paired end sequencing reads to a database of all UCSC knowngene exon-exon junctions and all other expressed circular junctions (see Figure S5). The rate of enrichment averaged 1.05 and was never more than 1.25 per offset. While this analysis does not strictly consider all microRNA binding sites within a circle, it samples a window including circular RNA sequence that, under most basic models where circular RNA were under selection to serve as a microRNA sink, would be enriched for microRNA seed matches.

## Supporting Information

Figure S1 Intron length as fraction of maximum length around exons defining circular RNA. Intron lengths flanking circular isoforms are calculated as described in the main text. A) and B) show the genome-wide distributions of flanking intron length, normalized by their ratio to the maximum intron length within a gene; A) weights each isoform by total reads summed over all replicates and samples; B) counts each isoform once, provided it has at least 20 distinct read counts.
(EPS)

Figure S2 Linear Isoform variation does not predict circular isoform variation: Bars are colored by isoform per gene. Linear isoform-specific expression for LPAR1, RNF19B, ZFAND6 per cell type does not show a relationship to circular isoform variation. Colors of bars represent different GENCODE V7 isoforms and are consistent across cell types within a gene. Linear isoform expression in LPAR1 is similar between NHLF and AG04450 (sharing the same dominant isoform), but different from BJ and HSMM, which have a different dominant isoform. Circular isoform expression is similar between the pairs BJ and AG04450 and different from the similar pair NHLF and HSMM). The same trend holds for RNF19B: HMEC and NHEK have the same dominant linear isoform (different from all other cell types), whereas NHLF has a distinct circular isoform expression pattern. No alternative linear isoforms are annotated for ZFAND6. Data was taken from analysis available from ENCODE poly(A)+ transcript quantification with a GENCODE V7 annotation.
(EPS)

Figure S3 Number of detected circular isoforms correlates with total circular isoform expression. Across cell types and replicates, total sequencing counts representing all detected circles on the x axis and number of distinct isoforms on the y axis are correlated. However, for a fixed level of circle expression, greater variation in number of distinct isoforms is observed across cell types than replicates.
(EPS)

Figure S4 Combinatorial features of circular RNA isoform expression. A): A genome-wide distribution of circle expression (x axis) vs number of detected circles (y axis) showing that the most highly expressed circles also exhibit the largest number of detectable circular isoforms. B) numbers of splice acceptor and donor sites used in circle splicing are correlated. C) genome-wide combinatorial index is low: most loci only express a small subset of circles compared to all possible splice site pairings. Moreover, increased total expression of circular isoforms does not show a relationship with increased detection of circles involving all potential splice site pairs, measured by the combinatorial index.
(TIF)

Figure S5 No systematic enrichment for microRNA sites near circle junctions. The only sequences unique to circles (not present in linear counterparts) are in the region of the scrambled exon-exon junction. We test if microRNA seed matches (determined using mature.fa from http://www.mirbase.org/ftp.shtml) might be enriched in a 66 nt window around these junctions (the junction is at offset position 66). At each offset position, we plot the ratio of microRNA seed matches in highly expressed circles (rank <1000 in at least one cell type) to lowly expressed circles (all others). The average ratio was roughly 1.05 over all offsets, with a maximum of 1.25 at any position.
(TIFF)

Table S1 Expression of circular and linear isoforms and normalized relative expression for A549. qPCR was performed as described as in the main text to normalize between linear and circular RNA expression measurements. Genes are ordered according to circular: linear ratios which is provided in the field rpkm_div_cir.count. The value at which rpkm_div_cir.count corresponds to equal numbers of linear and circular molecules is 315.05. cir.count is the number of total RNA-Seq reads representing each circular junction.
(CSV)

Table S2 Expression of circular and linear isoforms and normalized relative expression for AG04450. qPCR was performed as described as in the main text to normalize between linear and circular RNA expression measurements. Genes are ordered according to circular: linear ratios and provided in the field rpkm_div_cir.count. The value at which rpkm_div_cir.count corresponds to equal numbers of linear and circular molecules is 523.942. cir.count is the number of total RNA-Seq reads representing each circular junction.
(CSV)

Table S3 Expression of circular and linear isoforms and normalized relative expression for HeLa-S3. qPCR was performed as described as in the main text to normalize between linear and circular RNA expression measurements. Genes are ordered according to circular: linear ratios which is provided in the field rpkm_div_cir.cout. The value at which rpkm_div_cir.cout corresponds to equal numbers of linear and circular molecules is 275.005. cir.count is the number of total RNA-Seq reads representing each circular junction.
(CSV)

Table S4 Estimated .999 CI of rate of donor site used in expressed circular RNA by ENCODE cell type per circular isoform. Fields in table: type, method = exact binomial CI, x = number of circles using donor site per gene per cell type, n = total number of circles in this gene and type, mean = fraction of circles using this donor site, lower = lower .999 CI for p, upper = upper .999 CI for p, donor = donor position, gene.
(CSV)

Table S5 Estimated .999 CI of rate of acceptor site used in expressed circular RNA by ENCODE cell type per circular isoform. Fields in table: type, method = exact binomial CI, x = number of circles using acceptor site per gene per cell type, n = total number of circles in this gene and type, mean = fraction of circles using this acceptor site, lower = lower .999 CI for p, upper = upper .999 CI for p, acceptor = acceptor position, gene.
(CSV)

Table S6 All circular junctions detected in the *Drosophila* data. SRR = data file id; gene = common gene name; flygene_id = flygene identifier; exon1 = acceptor exon–exons enumerated beginning with 0; exon2 = donor exon (exons enumerated beginning with 0); strain = period null or wt; time = time of replicate; count = total circle counts.
(CSV)

Table S7 All circular junctions detected in the ENCODE data. Fields in table: type = cell line; place = location of RNA isolate given by ENCODE; start = acceptor (donor) site and stop = donor (acceptor) site for detected circular junction for genes transcribed on the “+” (resp. “−”) strand;ave_size = average length of all transcripts in UCSC knowngene annotation between start and stop coordinates; gene; score1info,score2info,score12info = average alignment score for read 1, read 2 and product of read 1 and read 2 (respectively),sum = number of junctional counts; srr = replicate, rank = ranked circle expression (highest = 1).
(BZ2)

Table S8 qPCR primers for linear and circular isoform detection.
(DOCX)

Table S9 Estimated coefficients in Poisson model of linear and circular isoform abundance by gene. Fields in table: reptype = celltype and replicate type, celltype = cell type, isomark = concatenated coordinates of start and stop from Table S1, genexp = RPKM of linear transcript,circle = circle counts, gene, logrpkm = log (rpmk) gene expression, resid = residual from fitting the GLM with poisson link in R below using the first 6 columns, fitted = fitted value after fitting the GLM with poisson link in R below using the first 6 columns: gg = glm(as.numeric(as.vector(rall$circle))∼log(as.numeric(as.vector(rall$genexp))) * rall$iso+rall$reptype, family = “poisson”).
(CSV)

## References

[1] Salzman J, Gawad C, Wang PL, Lacayo N, Brown PO (2012) Circular RNAs are the predominant transcript isoform from hundreds of human genes in diverse cell types. PloS one 7: e30733. 22319583 10.1371/journal.pone.0030733 PMC3270023

[2] Capel B, Swain A, Nicolis S, Hacker A, Walter M, et al (1993) Circular transcripts of the testis-determining gene Sry in adult mouse testis. Cell 73: 1019–1030. 7684656 10.1016/0092-8674(93)90279-y

[3] Houseley JM, Garcia-Casado Z, Pascual M, Paricio N, O'Dell KM, et al (2006) Noncanonical RNAs from transcripts of the Drosophila muscleblind gene. The Journal of heredity 97: 253–260. 16714427 10.1093/jhered/esj037

[4] Zaphiropoulos PG (1996) Circular RNAs from transcripts of the rat cytochrome P450 2C24 gene: correlation with exon skipping. Proceedings of the National Academy of Sciences of the United States of America 93: 6536–6541. 8692851 10.1073/pnas.93.13.6536 PMC39059

[5] Bailleul B (1996) During in vivo maturation of eukaryotic nuclear mRNA, splicing yields excised exon circles. Nucleic acids research 24: 1015–1019. 8604331 10.1093/nar/24.6.1015 PMC145744

[6] Burd CE, Jeck WR, Liu Y, Sanoff HK, Wang Z, et al (2010) Expression of linear and novel circular forms of an INK4/ARF-associated non-coding RNA correlates with atherosclerosis risk. PLoS genetics 6: e1001233. 21151960 10.1371/journal.pgen.1001233 PMC2996334

[7] Jeck WR, Sorrentino JA, Wang K, Slevin MK, Burd CE, et al (2013) Circular RNAs are abundant, conserved, and associated with ALU repeats. RNA 19: 141–157. 23249747 10.1261/rna.035667.112 PMC3543092

[8] Memczak S, Jens M, Elefsinioti A, Torti F, Krueger J, et al (2013) Circular RNAs are a large class of animal RNAs with regulatory potency. Nature 495 7441: 333–8. 23446348 10.1038/nature11928

[9] Hansen TB, Jensen TI, Clausen BH, Bramsen JB, Finsen B, et al (2013) Natural RNA circles function as efficient microRNA sponges. Nature 495: 384–388. 23446346 10.1038/nature11993

[10] Vincent HA, Deutscher MP (2006) Substrate recognition and catalysis by the exoribonuclease RNase R. The Journal of biological chemistry 281: 29769–29775. 16893880 10.1074/jbc.M606744200

[11] Pak J, Maniar JM, Mello CC, Fire A (2012) Protection from feed-forward amplification in an amplified RNAi mechanism. Cell 151: 885–899. 23141544 10.1016/j.cell.2012.10.022 PMC3499135

[12] Diener TO (1971) Potato spindle tuber virus: a plant virus with properties of a free nucleic acid. 3. Subcellular location of PSTV-RNA and the question of whether virions exist in extracts or in situ. Virology 43: 75–89. 5543290 10.1016/0042-6822(71)90226-1

[13] Versteeg R, van Schaik BD, van Batenburg MF, Roos M, Monajemi R, et al (2003) The human transcriptome map reveals extremes in gene density, intron length, GC content, and repeat pattern for domains of highly and weakly expressed genes. Genome research 13: 1998–2004. 12915492 10.1101/gr.1649303 PMC403669

[14] King AN, Beer DG, Christensen PJ, Simpson RU, Ramnath N (2010) The vitamin D/CYP24A1 story in cancer. Anti-cancer agents in medicinal chemistry 10: 213–224. 20184548 10.2174/1871520611009030213

[15] Horvath HC, Lakatos P, Kosa JP, Bacsi K, Borka K, et al (2010) The candidate oncogene CYP24A1: A potential biomarker for colorectal tumorigenesis. The journal of histochemistry and cytochemistry : official journal of the Histochemistry Society 58: 277–285. 19901270 10.1369/jhc.2009.954339 PMC2825493

[16] Horvath E, Lakatos P, Balla B, Kosa JP, Tobias B, et al (2012) Marked Increase of CYP24A1 mRNA Level in Hepatocellular Carcinoma Cell Lines Following Vitamin D Administration. Anticancer research 32: 4791–4796. 23155244

[17] Chen G, Kim SH, King AN, Zhao L, Simpson RU, et al (2011) CYP24A1 is an independent prognostic marker of survival in patients with lung adenocarcinoma. Clinical cancer research : an official journal of the American Association for Cancer Research 17: 817–826. 21169243 10.1158/1078-0432.CCR-10-1789 PMC3058389

[18] Balla B, Kosa JP, Tobias B, Halaszlaki C, Takacs I, et al (2011) Marked increase in CYP24A1 gene expression in human papillary thyroid cancer. Thyroid : official journal of the American Thyroid Association 21: 459–460. 21385079 10.1089/thy.2010.0420

[19] Anderson MG, Nakane M, Ruan X, Kroeger PE, Wu-Wong JR (2006) Expression of VDR and CYP24A1 mRNA in human tumors. Cancer chemotherapy and pharmacology 57: 234–240. 16180015 10.1007/s00280-005-0059-7

[20] Cerami E, Gao J, Dogrusoz U, Gross BE, Sumer SO, et al (2012) The cBio cancer genomics portal: an open platform for exploring multidimensional cancer genomics data. Cancer discovery 2: 401–404. 22588877 10.1158/2159-8290.CD-12-0095 PMC3956037

[21] Kroeldrup L, Kjaergaard S, Kirchhoff M, Kock K, Brasch-Andersen C, et al (2012) Duplication of 7q36.3 encompassing the Sonic Hedgehog (SHH) gene is associated with congenital muscular hypertrophy. European journal of medical genetics 55: 557–560. 22683912 10.1016/j.ejmg.2012.04.009

[22] Fimia GM, Corazzari M, Antonioli M, Piacentini M (2012) Ambra1 at the crossroad between autophagy and cell death. Oncogene 32: 3311–8. 23069654 10.1038/onc.2012.455

[23] Fimia GM, Stoykova A, Romagnoli A, Giunta L, Di Bartolomeo S, et al (2007) Ambra1 regulates autophagy and development of the nervous system. Nature 447: 1121–1125. 17589504 10.1038/nature05925

[24] Graveley BR, Brooks AN, Carlson JW, Duff MO, Landolin JM, et al (2011) The developmental transcriptome of Drosophila melanogaster. Nature 471: 473–479. 21179090 10.1038/nature09715 PMC3075879

[25] Hsu MT, Coca-Prados M (1979) Electron microscopic evidence for the circular form of RNA in the cytoplasm of eukaryotic cells. Nature 280: 339–340. 460409 10.1038/280339a0

[26] Dong LM, Ulrich CM, Hsu L, Duggan DJ, Benitez DS, et al (2009) Vitamin D related genes, CYP24A1 and CYP27B1, and colon cancer risk. Cancer epidemiology, biomarkers & prevention : a publication of the American Association for Cancer Research, cosponsored by the American Society of Preventive Oncology 18: 2540–2548. 10.1158/1055-9965.EPI-09-0228 PMC2761078 19706847

[27] Fang Z, Xiong Y, Zhang C, Li J, Liu L, et al (2010) Coexistence of copy number increases of ZNF217 and CYP24A1 in colorectal cancers in a Chinese population. Oncology letters 1: 925–930. 22966406 10.3892/ol_00000163 PMC3436466

[28] Hobaus J, Fetahu IS, Khorchide M, Manhardt T, Kallay E (2012) Epigenetic regulation of the 1,25-dihydroxyvitamin D(3) 24-hydroxylase (CYP24A1) in colon cancer cells. The Journal of steroid biochemistry and molecular biology 136: 296–299. 22940288 10.1016/j.jsbmb.2012.08.003 PMC3695570

[29] Holick CN, Stanford JL, Kwon EM, Ostrander EA, Nejentsev S, et al (2007) Comprehensive association analysis of the vitamin D pathway genes, VDR, CYP27B1, and CYP24A1, in prostate cancer. Cancer epidemiology, biomarkers & prevention: a publication of the American Association for Cancer Research, cosponsored by the American Society of Preventive Oncology 16: 1990–1999. 10.1158/1055-9965.EPI-07-0487 17932346

[30] Horvath HC, Khabir Z, Nittke T, Gruber S, Speer G, et al (2010) CYP24A1 splice variants–implications for the antitumorigenic actions of 1,25-(OH)2D3 in colorectal cancer. The Journal of steroid biochemistry and molecular biology 121: 76–79. 20398751 10.1016/j.jsbmb.2010.03.080

[31] Lopes N, Sousa B, Martins D, Gomes M, Vieira D, et al (2010) Alterations in Vitamin D signalling and metabolic pathways in breast cancer progression: a study of VDR, CYP27B1 and CYP24A1 expression in benign and malignant breast lesions. BMC cancer 10: 483. 20831823 10.1186/1471-2407-10-483 PMC2945944

[32] Zeljic K, Supic G, Stamenkovic Radak M, Jovic N, Kozomara R, et al (2012) Vitamin D receptor, CYP27B1 and CYP24A1 genes polymorphisms association with oral cancer risk and survival. Journal of oral pathology & medicine : official publication of the International Association of Oral Pathologists and the American Academy of Oral Pathology 41: 779–787. 10.1111/j.1600-0714.2012.01164.x 22612324

[33] Chen CY, Sarnow P (1995) Initiation of protein synthesis by the eukaryotic translational apparatus on circular RNAs. Science 268: 415–417. 7536344 10.1126/science.7536344

[34] Chen CY, Sarnow P (1998) Internal ribosome entry sites tests with circular mRNAs. Methods in molecular biology 77: 355–363. 9770681 10.1385/0-89603-397-X:355

[35] Langmead B, Salzberg SL (2012) Fast gapped-read alignment with Bowtie 2. Nature Methods 9: 357–359. 22388286 10.1038/nmeth.1923 PMC3322381

[36] Burnett WV (1997) Northern blotting of RNA denatured in glyoxal without buffer recirculation. BioTechniques 22: 668–671. 9105618 10.2144/97224st01

