# Correction: Cell-Type Specific Features of Circular RNA Expression

**DOI:** 10.1371/annotation/f782282b-eefa-4c8d-985c-b1484e845855

**Published:** 2013-12-30

**Authors:** Julia Salzman, Raymond E. Chen, Mari N. Olsen, Peter L. Wang, Patrick O. Brown

Patrick O Brown should also be listed as a corresponding author. His email address is pbrown@stanford.edu

