# Supplementary figures and images for: Cell-Type Specific Features of Circular RNA Expression
Source: PLoS Genet. 2013 Sep 5;9(9):e1003777. doi: 10.1371/journal.pgen.1003777 (PMC3764148; doi:10.1371/journal.pgen.1003777)

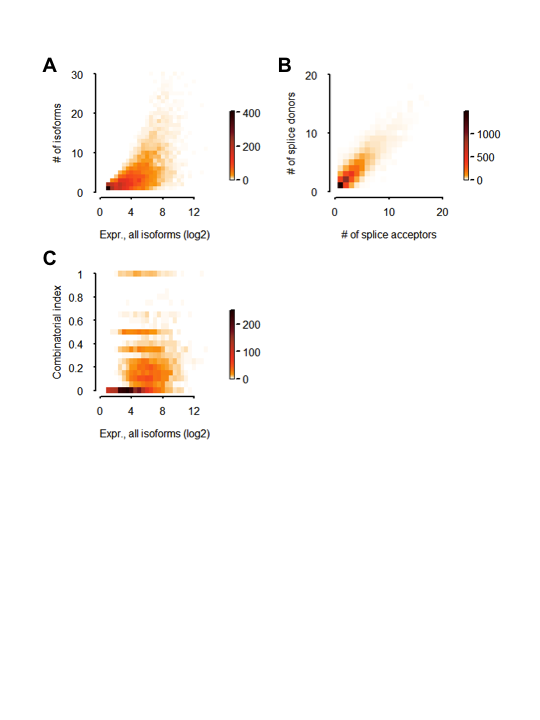

Supplement: Figure S4 — Combinatorial features of circular RNA isoform expression. A): A genome-wide distribution of circle expression (x axis) vs number of detected circles (y axis) showing that the most highly expressed circles also exhibit the largest number of detectable circular isoforms. B) numbers of splice acceptor and donor sites used in circle splicing are correlated. C) genome-wide combinatorial index is low: most loci only express a small subset of circles compared to all possible splice site pairings. Moreover, increased total expression of circular isoforms does not show a relationship with increased detection of circles involving all potential splice site pairs, measured by the combinatorial index. (TIF) [file pgen.1003777.s004.tif]

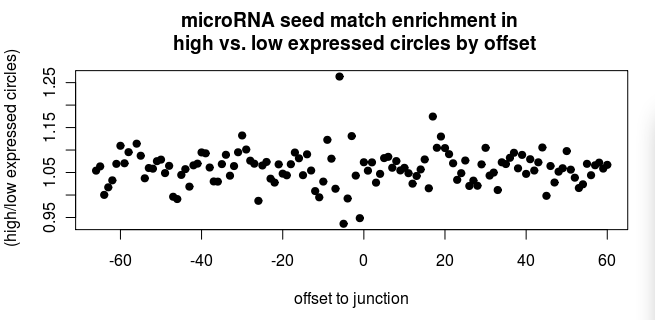

Supplement: Figure S5 — No systematic enrichment for microRNA sites near circle junctions. The only sequences unique to circles (not present in linear counterparts) are in the region of the scrambled exon-exon junction. We test if microRNA seed matches (determined using mature.fa from http://www.mirbase.org/ftp.shtml) might be enriched in a 66 nt window around these junctions (the junction is at offset position 66). At each offset position, we plot the ratio of microRNA seed matches in highly expressed circles (rank <1000 in at least one cell type) to lowly expressed circles (all others). The average ratio was roughly 1.05 over all offsets, with a maximum of 1.25 at any position. (TIFF) [file pgen.1003777.s005.tiff]
