# Supplementary material for: Cell-Type Specific Features of Circular RNA Expression
Source: PLoS Genet. 2013 Sep 5;9(9):e1003777. doi: 10.1371/journal.pgen.1003777 (PMC3764148; doi:10.1371/journal.pgen.1003777)
Supplement: Table S8 — qPCR primers for linear and circular isoform detection. (DOCX) [file pgen.1003777.s013.docx]

> ABTB1-lin-probe

TG GTT CTC AG GCA CCC ACT GAT

> ABTB1-lin-fwd

CCA ACA TGC TGG ACA CCA AATGGA

> ABTB1-lin-rev

AGT CAC TCA CAT GCT CTA CGCCAA

> ABTB1-cir-probe

AGCTGGTACTCTACCTTCTGGCCAAT

> ABTB1-cir-fwd

TACTATGCCTGCTTGTGTGGGCA

> ABTB1-cir-rev

TCCACGTCTCGCTGCTCCAG

> CYP24A1-cir-probe

AA ATC AAT GA GGT CTT GGC CAG GCC A

> CYP24A1-cir-fwd

GGG AAG TGA TGA AGC TGG ACA ACA

> CYP24A1-cir-rev

CTG AGG CGT ATT ATC GCT GGC AAA

> CYP24A1-lin-probe

GG TGG AGT AC CAC AAG AAG TAT GGC AAG

> CYP24A1-lin-fwd

TCT CAA GAA ACA GCA CGA CAC CCT

> CYP24A1-lin-rev

CAA AGG AAC CCA ACT TCA TGC GGA

> HIPK3-cir-probe

AC AGG TAT GG CCT CAC AAG TCT TGG T

> HIPK3-cir-F

CTG AGT TTG ATA AAC ATA TGG TGG GTA G

> HIPK3-cir-R

CTG TTT GTT CAA CAT ATC TAC AAT CTC GG

> HIPK3-lin -probe

TT ACT CCA GC TGA GAC CCT GAA CCA

> HIPK3-lin -F

TGG TTG CTA TGA GGG AAA TCT AGA AG

> HIPK3- lin -R

GTG AAC ACA GTG ATG GAT TTG GAA GG

> FAT1-cir-probe

TC GAA GAC GG GAA TGA GCA TGG CAA

> FAT1-cir-F

AAA TCA GAC TCC CTG AGC GGG AAA

> FAT1-cir-R

AAC CTC TTG GAC GAA ACC ACT CCA

> FAT1-lin-probe

TT TGG CTT TG CTC CTG CTT CTG CTC CTT

> FAT1-lin-F

ATT AGA GAT GGC TCT GGC GTT GGT

> FAT1-lin-R

AGG AGT CTG TTC AAG TCG TTG GCT

>LPAR1-cir-probe

AA GTA GAG AT GGC AGC CAT GAC AGC

>LPAR1-cir-F

GCG TGT TCA CCA CCT ACA A

>LPAR1-cir-R

TGA GAA GTC AGG TAC TCA GAT AGG

>LPAR1-lin-probe

AT GGA ACA CA GTC AGC AAG CTG GT

>LPAR1-lin-F

ACG AGT CCA TTG CCT TCT TT

>LPAR1-lin-R

ACA GTG ATT CCA AGT CCC ATC

>LINC00340-cir-probe

TG GAT CTG CA TTT ACT GCT CAA CCA CA

>LINC00340-cir-F

GAC AGA AGA AGG AAG TGG CTA A

>LINC00340-cir-R

GTA GTA ACC CAG GGA TGA CTT G

>LINC00340-lin-probe

TG CGT TGT TC TTA CTG GAT CTT TCC ATC A

>LINC00340-lin-F

CTA GCA CTG ACC TCC TTC ATT C

>LINC00340-lin-R

TCC AGG AAT GAT TTG ACC TTG TA

>PVT1-cir-probe

TG GTG AAG CA TCT GAT GCA CGT TCC A

>PVT1-cir-F

AGC TGA CAG GCA CAG CCA TCT TGA

>PVT1-cir-R

CCT TTG GGT CTC CCT ATG GAA TGT

>PVT1-lin-probe

TT GGC ACG TG GCT CCC TTG GTG TT

>PVT1-cir-F

TCT CCA AAT CTC AGT GTC CTG GCA

>PVT1-cir-R

TGA CCT TGG CAC ATA CAG CCA TCA

>RNF220-cir-probe

CAA CCA GCACTT TGG GTC AGG G

>RNF220-cir-F

CCA TCG GAG TCT CTT TCT GTG

>RNF220-cir-R

CAG AGG ATC GGA ATG ACA GAG

>RNF220-lin-probe

AC CGC TTT GA GGA GTA TGA GTG GTG T

>RNF220-lin-F

ATG CTG TGG ACA TCG AGC ATG AGA

>RNF220-lin-R

AGC ATC ACT GTC CGG GTT CTC TTT
